# Insights Into the Role of Copper in Neurodegenerative Diseases and the Therapeutic Potential of Natural Compounds

**DOI:** 10.2174/1570159X22666231103085859

**Published:** 2023-11-15

**Authors:** Guangcheng Zhong, Xinyue Wang, Jiaqi Li, Zhouyuan Xie, Qiqing Wu, Jiaxin Chen, Yiyun Wang, Ziying Chen, Xinyue Cao, Tianyao Li, Jinman Liu, Qi Wang

**Affiliations:** 1 Science and Technology Innovation Center, Guangzhou University of Chinese Medicine, Guangzhou, China;; 2 The Sixth Affiliated Hospital, Sun Yat-sen University, Guangzhou, China;; 3 Affiliated Jiangmen TCM Hospital of Ji'nan University, Jiangmen, China

**Keywords:** Neurodegenerative diseases, cognitive impairments, copper chelators, metal-protein attenuating compounds, natural compounds, polyphenol

## Abstract

Neurodegenerative diseases encompass a collection of neurological disorders originating from the progressive degeneration of neurons, resulting in the dysfunction of neurons. Unfortunately, effective therapeutic interventions for these diseases are presently lacking. Copper (Cu), a crucial trace element within the human body, assumes a pivotal role in various biological metabolic processes, including energy metabolism, antioxidant defense, and neurotransmission. These processes are vital for the sustenance, growth, and development of organisms. Mounting evidence suggests that disrupted copper homeostasis contributes to numerous age-related neurodegenerative diseases, such as Alzheimer's disease (AD), Parkinson's disease (PD), Huntington's disease (HD), amyotrophic lateral sclerosis (ALS), Wilson's disease (WD), Menkes disease (MD), prion diseases, and multiple sclerosis (MS). This comprehensive review investigates the connection between the imbalance of copper homeostasis and neurodegenerative diseases, summarizing pertinent drugs and therapies that ameliorate neuropathological changes, motor deficits, and cognitive impairments in these conditions through the modulation of copper metabolism. These interventions include Metal-Protein Attenuating Compounds (MPACs), copper chelators, copper supplements, and zinc salts. Moreover, this review highlights the potential of active compounds derived from natural plant medicines to enhance neurodegenerative disease outcomes by regulating copper homeostasis. Among these compounds, polyphenols are particularly abundant. Consequently, this review holds significant implications for the future development of innovative drugs targeting the treatment of neurodegenerative diseases.

## 1 INTRODUCTION

Neurodegenerative diseases are a heterogeneous group of diseases with severe dysfunction characterized by selective and progressive loss of specific neuronal functions [1]. Numerous studies have demonstrated that, despite the clinical symptoms of most neurological diseases being different, they share specific molecular and pathological features, such as oxidative stress [2], autophagy impairment [3], protein misfolding, and abnormal aggregation [4, 5]. Among the critical factors in various age-related neurodegenerative diseases is the breakdown of metal homeostasis in brain tissue [2]. In particular, it is closely related to the metabolic dysregulation of copper (Cu) in the brain [6, 7].

Copper occurs naturally in soil and groundwater and is an electrically conductive and ductile metal used throughout the world for centuries. Foods such as animal liver, nuts, legumes, fish, shrimp, and shellfish contain high levels of copper, whereas dairy products contain low levels [8]. For example, sheep liver contains 157 mg/kg copper, lobster contains 36.6 mg/kg copper [9], nuts such as hazelnuts and cashew nuts contain 14.8-22.5 mg/kg copper [10], while dairy products such as milk, yogurt, and cheese contain about 50-120 μg/L of copper [11]. Under physiological conditions, the body of a healthy adult contains approximately 110 mg of copper, of which approximately two-thirds are distributed in bones and muscles. Moreover, copper is primarily distributed in the liver (10 mg), brain (8.8 mg), and blood (6 mg) [12]. As one of the transition metal elements necessary for the human body, copper acts as an electron acceptor or donor by converting between its two oxidation states, Cu^+^ (organic copper) and Cu^2+^ (inorganic copper), thereby participating in various reactions and playing a significant role [8, 13]. Copper is a fundamental structural component and essential cofactor of many enzymes, including copper/zinc-superoxide dismutase (Cu/Zn SOD or SOD1) (antioxidant defense), ceruloplasmin (Cp) (iron metabolism), cytochrome c oxidase (CCO) (energy metabolism), tyrosinase (pigmentation), peptidylglycine-α-amidating enzyme (neuropeptide synthesis) and dopamine-β-monooxygenase (neurotransmission), all of which are dependent on copper for their catalytic activity [14-16]. Furthermore, copper is involved in many biological processes, including angiogenesis, connective tissue formation, catecholamine biosynthesis, and myelination [17, 18]. The body maintains copper levels within the normal range through precise homeostatic control mechanisms, including copper's absorption, transport, and excretion. Intracellular copper metabolism must be tightly controlled since elevated or decreased copper levels may cause neurotoxicity through different mechanisms. For example, when copper is overloaded, its redox activity enables it to catalyze the production of reactive oxygen species (ROS), including hydroxyl radicals (OH^-^), superoxide anions (O_2_^-^), and hydrogen peroxide (H_2_O_2_) through Fenton and Haber-Weiss reactions, which leads to oxidative damage to proteins, lipids, and deoxyribonucleic acid (DNA), resulting in neuronal dysfunction and cell death [8, 14, 17]. There is evidence that excessive copper contributes to the pathogenesis of Alzheimer's disease (AD), Parkinson's disease (PD), amyotrophic lateral sclerosis (ALS), multiple sclerosis (MS), and Wilson's disease (WD) [17]. Copper deficiency reduces the body's ability to scavenge free radicals because copper acts as a cofactor in many antioxidant enzymes [19]. In addition, in the nervous system, copper plays a key role in a variety of neuronal functions by regulating synaptic activity, neurotrophic-induced signaling cascades, and excitotoxic cell death, and is also closely related to the occurrence and development of AD, PD, and Menkes disease (MD) [17]. The recently reported cuproptosis also revealed the importance of copper homeostasis, which is a novel form of cell death that occurs through the direct binding of copper to lipoylated components of the tricarboxylic acid (TCA) cycle. The aggregation of these copper-bound lipoylated mitochondrial proteins and the subsequent loss of iron-sulfur (Fe-S) cluster proteins induce proteotoxic stress and, ultimately, cell death [20, 21]. Given that several neurodegenerative diseases are strongly associated with abnormal copper homeostasis, correcting the disturbed copper homeostasis in the body and brain by copper chelation or supplementation may be a promising strategy for treating neurodegenerative diseases.

**Fig. (1):**
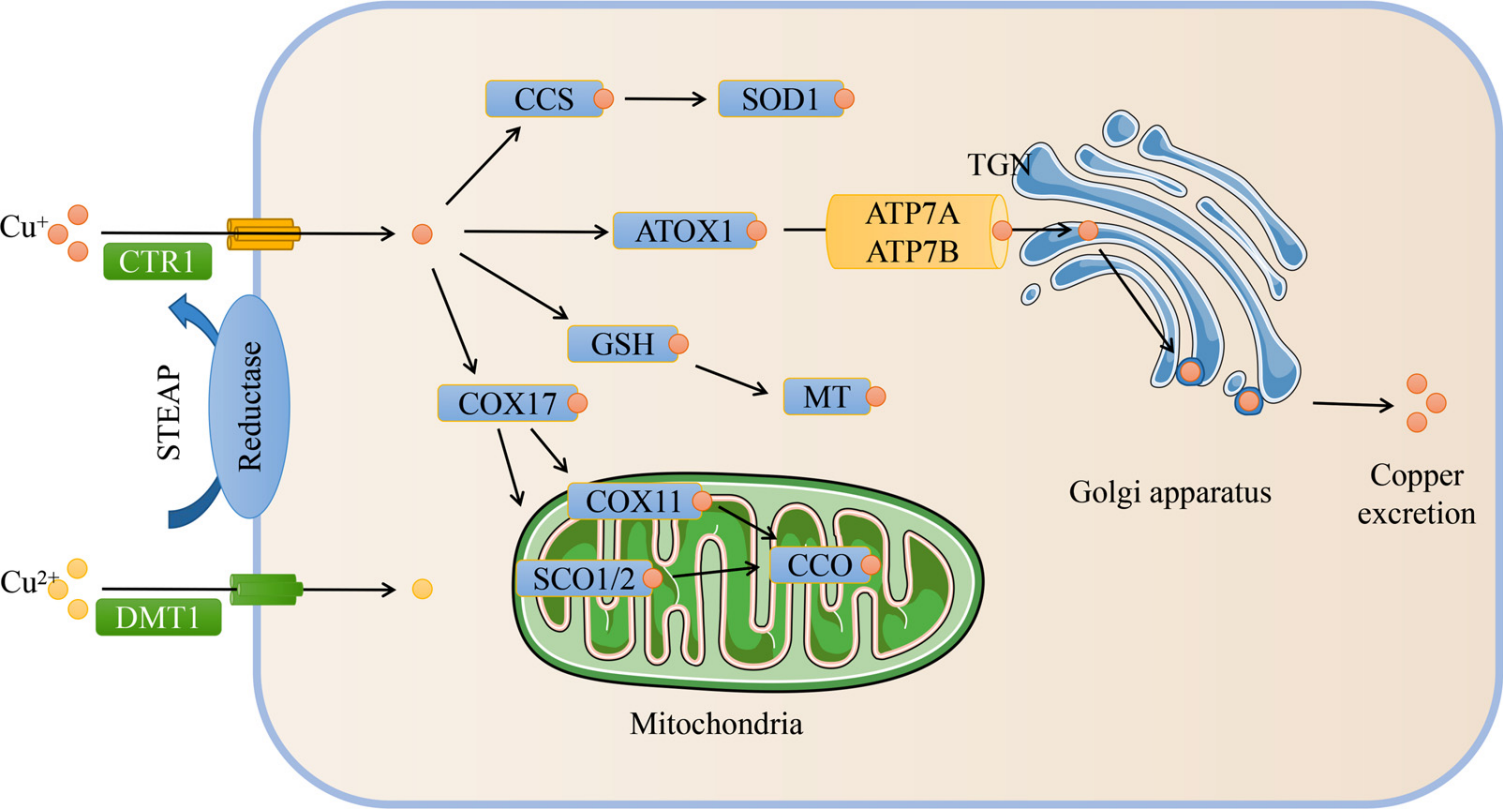
Schematic diagram of the mechanism of cellular copper uptake, distribution, and metabolism. Extracellularly, Cu^2+^ is reduced to Cu^+^ by reductases such as the six transmembrane epithelial antigen of the prostate (STEAP) and then transported into the cell through the membrane protein CTR1 [27, 28], and delivered to specific sites by several copper transporters, including ATOX1, CCS, and COX17. In addition, DMT1 on the plasma membrane can directly incorporate Cu^2+^ into the cell [13]. ATOX1 transports copper to ATP7A and ATP7B located in the trans-Golgi network (TGN) and subsequently binds to various copper-dependent enzymes [12]. To facilitate the excretion of excess intracellular copper, ATPases are translocated from the TGN to the plasma membrane when intracellular copper levels are elevated [28]. CCS incorporates copper into cytoplasmic SOD1, which catalyzes the dismutation of O_2_.^−^ into H_2_O_2_ and molecular oxygen, protecting cells from free radical damage [28]. COX17 transports copper into COX11, SCO1, and SCO2 in the mitochondria and subsequently binds to CCO for its metallation and activation [29]. In addition, intracellular copper also binds to glutathione (GSH) and mediates copper transport to metallothionein (MT) for storage [26].

**Fig. (2):**
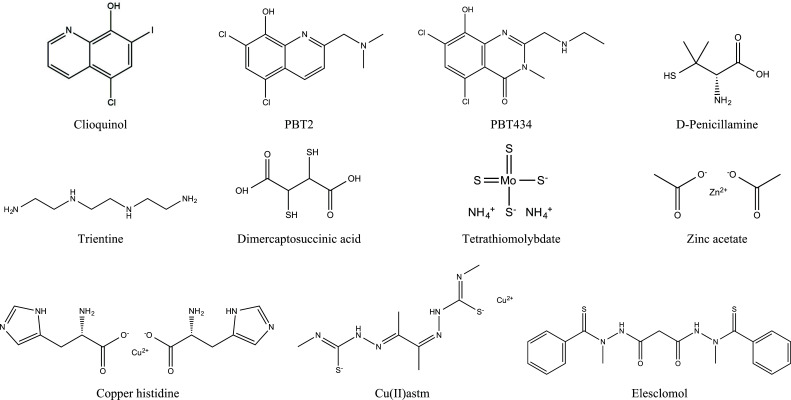
Chemical structures of several drugs targeting copper for the treatment of neurodegenerative diseases. CQ is used in the treatment of AD, PD, HD, and MS; PBT2 is used in the treatment of AD and HD; PBT434 is used in the treatment of PD; DPA is used in the treatment of AD and WD; Trientine, DMSA and zinc acetate are used in the treatment of WD; TTM is used in the treatment of ALS and WD; Copper-histidine and elesclomol are used in the treatment of MD; CuII (atsm) is used in the treatment of PD and ALS.

Traditional Chinese Medicine (TCM) has long been used in the treatment and prevention of neurodegenerative diseases. It has unique advantages and potential for neurodegenerative diseases with complex pathogenesis due to its multi-component, multi-target, and multi-functional characteristics [22, 23]. In this paper, we reviewed the function and metabolism of copper (Fig. **1**) and analyzed the potential relationship between copper metabolism and several neurodegenerative diseases (Table **1**), including AD, PD, Huntington's disease (HD), ALS, WD, MD, prion diseases, and MS. In addition, this review also summarizes related drugs and therapies that regulate copper metabolism (Fig. **2**) and natural compounds with the potential to improve neurodegenerative diseases by regulating copper metabolism (Tables **2**, **3**), providing a scientific and theoretical basis for drug development for the treatment of neurodegenerative diseases.

## 2 COPPER METABOLISM IN THE BODY

In the body, cellular copper transport requires various membrane copper transporters, including copper transporter 1 (CTR1, SLC31A1), divalent metal transporter 1 (DMT1), P-type copper-transporting ATPase α (ATP7A) and β (ATP7B), as well as various copper chaperones, including antioxidant protein 1 (ATOX1), copper chaperone for CCO (COX17), and copper chaperone for superoxide dismutase (CCS). They are responsible for transporting copper across cell membranes and delivering it to specific intracellular targets, ensuring copper homeostasis within the body by precisely regulating intracellular copper levels [12] (Fig. **1**). Ceruloplasmin is the main protein that carries copper in the blood, and it carries about 95% of the copper in plasma [24], while the remainder is bound to albumin, transcuprein, and amino acids, called “free copper,” also known as non-ceruloplasmin-bound copper (Non-Cp-Cu) [25]. Dietary copper is absorbed into the body by enterocytes in the gastrointestinal tract and transported to the liver through portal circulation as free copper. In hepatocytes, copper is incorporated into ceruloplasmin, which transports copper from the liver to extrahepatic tissues, and excess copper is secreted into the bile and eventually excreted in the feces [26-29].

## 3 COPPER METABOLISM IN THE BRAIN

Copper concentration in the human brain is estimated to be 3.1 to 5.1 μg/g wet weight, but the distribution is not uniform. The highest copper content (11.40 ± 2.50 μg/g wet tissue weight) was found in the substantia nigra (SN), and high levels were also detected in the hippocampus, cerebellum, olfactory bulb, hypothalamus, and cortex [17, 30]. The homeostasis of the internal environment of brain tissue and central nervous system (CNS) is maintained by the coordinated action of the brain barrier system composed of the blood-brain barrier (BBB) and the blood-cerebrospinal fluid (CSF) barrier (BCB). The BBB is composed of tightly connected capillary endothelial cells of the brain that block harmful substances from entering the brain through the blood [31, 32]. In contrast, the BCB is located in the choroid plexus, a highly vascularized and polarized tissue in the roof of the ventricle whose primary function is to produce and secrete CSF [33]. In conclusion, the BBB is the primary route for copper entry into the brain, while the BCB is the primary route for copper efflux from the brain. Copper crosses the BBB as free copper ions and is transported from the blood circulation to the brain parenchyma, where it is released into the CSF, while the BCB returns copper from the CSF to the blood [15].

The copper transporters CTR1, ATP7A, and ATP7B are present in brain capillary endothelial cells and choroidal epithelial cells. They jointly regulate copper homeostasis in the brain and mediate copper influx into brain parenchyma and CSF. As the primary gatekeeper for copper entering the brain through brain capillary endothelial cells and choroid plexus epithelial cells, CTR1 is primarily located in the apical membrane of choroid plexus epithelial cells [26]. In the brain, astrocytes take up copper through CTR1 and sequester excess copper in MT and GSH complexes to protect cells from reactive oxygen and nitrogen species (ROS/RNS) [34]. Furthermore, DMT1 is an additional pathway for copper uptake in the brain [35]. ATP7A is expressed in endothelial cells of the BBB and facilitates the transport of copper in the blood across the basolateral membrane and into the extravascular space of the brain [36]. It is also strongly expressed in choroid plexus epithelial cells and mediates copper transport across the BBB and BCB [18]. In addition, excess copper can flow into the CSF, where it is taken up by CTR1 and DMT1 in the choroidal epithelial microvilli [12]. CTR1 and DMT1 mediate copper entry into neuronal cells after it reaches the CSF, then metallochaperones deliver copper to target pathways and ultimately participate in the metallation of copper enzymes [37], and ATP7A transports excess copper back into the blood from the CSF [33].

## 4 COPPER HOMEOSTASIS IN NEURODEGENERATIVE DISEASES

### 4.1 Alzheimer’s Disease

AD is the most common neurodegenerative disease [38] and is clinically characterized by memory impairment, progressive cognitive decline, and impaired executive function [31], affecting approximately 47 million people worldwide [39]. As one of the most prominent risk factors for AD, the size of the blood-free copper pool was significantly increased in AD patients (17.2 ± 5.9 μmol/L, n = 47) as compared to age-matched healthy controls (12.6 ± 2.5 μmol/L, n = 44) [40]. In addition, a positive correlation was found between the size of the blood-free copper pool and the degree of cognitive impairment, the speed of cognitive decline, and the risk of converting from mild cognitive impairment (MCI) to AD [13]. Autopsy analysis indicates that the copper content in amyloid plaques of AD patients (25.0 ± 7.8 μg/g, n = 9) is 5.7 times greater than in normal brains (4.4 ± 1.5 μg/g, n = 5) [41, 42]. In contrast, reduced copper levels in the brain have been found both in the hippocampal (AD: 12.6 ± 1.2 μg/g dry weight, n = 10; control:16.8 ± 0.9 μg/g, n = 11) and amygdala regions (AD:13.0 ± 1.5 μg/g, n = 10; control: 19.8 ± 1.5 μg/g, n = 11), which resulted in severe histopathological changes without significant changes in CSF copper levels [43, 44]. In addition, ceruloplasmin levels were significantly higher in most brain regions in patients (caudate: 1.29 ± 0.17 μg/g, putamen: 1.17 ± 0.07 μg/g, SN:0.62 ± 0.02 μg/g, hippocampus: 0.39 ± 0.05 μg/g, entorhinal cortex: 0.35 ± 0.04 μg/g, temporal cortex: 0.40 ± 0.05 μg/g, frontal cortex: 1.07 ± 0.07 μg/g, parietal cortex: 0.32 ± 0.03 μg/g, n = 12) compared to controls (caudate: 0.47 ± 0.05 μg/g, putamen:0.61 ± 0.14 μg/g, SN: 0.37 ± 0.07 μg/g, hippocampus:0.21 ± 0.02 μg/g, entorhinal cortex: 0.19 ± 0.02 μg/g, temporal cortex: 0.23 ± 0.01 μg/g, frontal cortex: 0.45 ± 0.11 μg/g, parietal cortex: 0.19 ± 0.02 μg/g, n = 7) [45, 46].

Amyloid precursor protein (APP), β-site APP cleaving enzyme 1 (BACE1), amyloid β (Aβ), and tubulin-associated unit (Tau) are proteins that bind copper and contribute to copper homeostasis in the brain. Animal experiments have shown that APP binds to Cu^2+^ and reduces it to Cu^+^. This conversion allows copper ions to be gradually cleared in the brain, which may explain the higher levels of serum copper and lower levels of brain copper in AD patients [43]. Mice overexpressing the APP gene had lower copper levels in the brain, whereas knocking out the APP gene showed the opposite result [47]. Copper binds to BACE1 and regulates its activity, thereby affecting the metabolism of APP [48]. Cu^2+^ binds to Aβ peptides with high affinity and increases the ratio of α-helix and β-sheet structures, which may be responsible for Aβ aggregation. The formed Cu-Aβ complex reduced the expression of low-density lipoprotein receptor-related protein-1 (LRP1) by activating microglia and promoting the release of inflammatory factors such as tumor necrosis factor-α (TNF-α), thus enhancing neuroinflammation and Aβ clearance disorder. ROS generated by the Cu-Aβ complex also led to oxidative damage of Aβ peptides, and the removal of Cu^2+^ from Aβ suppressed oxidative damage and reduced cell death [43]. Furthermore, copper promoted tau hyperphosphorylation and aggregation [49], whereas copper chelators reduced tau phosphorylation in SH-SY5Y cells [50]. Copper plays an essential role in regulating LRP1-mediated Aβ clearance. It promoted the downregulation of LRP1 in the brain capillaries in a mouse model of AD, partly due to the interaction of copper with LRP1 and cellular prions resulting in LRP1 nitrotyrosination and proteosomal degradation [51]. LRP1 is also known to bind tau and directly interact with Aβ, APP, and Apolipoprotein E4 (ApoE4), thereby regulating the production and clearance of Aβ [52]. The Apolipoprotein E (ApoE) allele is the genetic risk locus with the strongest association with AD, approximately a quarter of patients with sporadic AD carry the ApoE4 allele, and they are more susceptible to copper toxicity. ApoE2 and ApoE3 have copper-binding cysteine and significantly higher antioxidant activity than ApoE4, which has a lower capacity to bind copper due to the lack of copper-binding cysteine and is detrimental to Aβ clearance from the brain. Experimental studies confirmed that copper-induced Aβ aggregation was most pronounced when the ApoE4 allele was present and reduced the efflux of Aβ from the brain [26, 53]. Interestingly, genetic variants in the ATP7B gene are associated with a higher risk of AD since patients carrying mutants in the ATP7B gene have higher levels of free copper [54], and the ATP7B gene is also involved in sporadic AD [55].

### 4.2 Parkinson’s Disease

PD is the second most common neurodegenerative disease after AD and the neurological disease with the fastest-growing morbidity and mortality worldwide [56, 57]. It is characterized by resting tremor, muscle rigidity, bradykinesia, postural gait disturbance, and some non-motor symptoms [58]. Current evidence suggests that PD is associated with copper and iron homeostasis abnormalities. Reduced copper and increased iron content were found in the caudate nucleus (copper concentrations in PD: 0.36 ± 0.04 μg/g, n = 14; control: 0.63 ± 0.12 μg/g, n = 7) and SN (copper concentrations in PD: 0.60 ± 0.07 μg/g, n = 14; control: 0.86 ± 0.09 μg/g, n = 7) of PD patients [46, 59]. The main pathological changes in PD are the progressive loss of dopaminergic neurons in the substantia nigra pars compacta (SNpc) and the presence of Lewy bodies formed by abnormal aggregation of soluble α-synuclein (α-syn) [60]. Copper binds α-syn with high affinity, promoting its aggregation and increasing oxidative stress [61]. Furthermore, copper and iron possess similar physicochemical properties and regulate each other in metabolic processes. In PD, copper deficiency may lead to iron deposition in the brain by affecting the activity of DMT1 and ceruloplasmin, affecting redox homeostasis and damaging dopaminergic neurons [59]. At the same time, elevated iron content may also lead to decreased concentrations of copper and ceruloplasmin in the brain [62]. In an iron-deficient rat model, copper levels in the brain parenchyma, CSF, and choroid plexus were significantly elevated, and copper transport in the brain was nearly doubled. In contrast, copper transport was reduced by about half in the brains of iron-overloaded rats [31]. In conclusion, the balance of these trace elements is crucial for maintaining copper homeostasis in the body.

### 4.3 Huntington’s Disease

HD is a rare autosomal dominant neurodegenerative disorder characterized by progressive motor, cognitive, and psychiatric deterioration. HD is caused by abnormal expansion of the cytosine-adenine-guanine (CAG) repeat sequence in exon 1 of the huntingtin (HTT) gene, which encodes the production of polyglutamine (polyQ). The repeated expansion of the CAG sequence leads to misfolding and abnormal aggregation of polyQ, which are major drivers of the translational production of mutant huntingtin (mHTT) and its toxic properties [63]. Abnormally elevated copper concentrations have been reported in the brains of HD patients and HD mouse models compared to controls [64, 65]. Particularly, copper levels in the putamen of HD patients (657 ± 126 nmol/g dry weight human brain, n = 4) increased by 64% compared to control patients (399 ± 35 nmol/g, n = 9), while copper levels in the SN of HD patients (1061 ± 229 nmol/g, n = 10) increased by 68% compared to control patients (629 ± 56 nmol/g, n = 10) [65]. However, many studies have demonstrated an increase in copper levels in the brain of HD patients; some studies reported no change or even a decrease in copper levels [66, 67]. Copper binds to HTT variants with 17 to 68 glutamine residues, whereas neither iron nor zinc binds to this region. Copper promotes the stable aggregation of HTT, while copper chelators inhibit this process [68]. *In vitro* and *in vivo* experiments revealed that copper increased the aggregation and toxicity of polyQ. Furthermore, copper interacts with histidine residues in the 171 amino acid N-terminal fragment of HTT, which may affect HTT fibril formation and oligomerization [64]. Notably, copper is also closely associated with inhibiting lactate dehydrogenase (LDH) activity in HD, which is instrumental in regulating lactate levels and providing neurons with energy substrates. In HD transgenic mice, lactate levels are elevated, and LDH activity is decreased. However, intrastriatal delivery of the LDH inhibitor Oxamate to normal mice resulted in HD-like neurodegeneration, and copper inhibited the activity of LDH, thereby inducing neurodegeneration in HD [45, 69]. The evidence that copper affects HTT aggregation and conformation and regulates energy metabolism supports the involvement of abnormal copper metabolism in the pathogenesis of HD.

### 4.4 Amyotrophic Lateral Sclerosis

ALS (Lou Gehrig's disease) is a progressive paralytic disease and the third most common neurodegenerative disease after AD and PD. It is characterized by muscle atrophy and paralysis resulting from the loss of upper and lower motor neurons in the motor cortex, brainstem, and spinal cord. Patients die within 2 to 5 years of diagnosis due to respiratory failure [70, 71]. ALS patients have elevated levels of copper ions in the motor cortex (ALS: 25.1 μg/g, control: 19.8 μg/g, n = 3-8) and decreased levels of copper ions (ALS: 913.21 ± 165.55 μg/L, n = 28; control: 1020.17 ± 197.76 μg/L, n = 38) and ceruloplasmin (ALS:23.2 ± 6.3 μg/L, n = 27; control: 25.0 ± 4.2 μg/L, n = 26) in the serum [27, 72-74]. In contrast, there have also been reports of decreased or no change in serum and CSF copper levels in patients with ALS compared with controls [45]. About 20% of familial ALS (FALS) cases are caused by mutations in the SOD1 gene, and approximately 150 SOD1 mutations have been identified [75]. The SOD1 gene encodes Cu/Zn SOD, which catalyzes the dismutation of O_2_.^−^ into H_2_O_2_ and molecular oxygen, protecting cells from free radical damage and thus acting as an antioxidant enzyme [76]. SOD1 is a metalloprotein that forms highly stable homodimers by binding copper and zinc ions [77]. Mutations in SOD1 affect metal binding and may be defective in binding copper and zinc [78]. Dissociation of copper and/or zinc ions significantly lowers the melting temperature of SOD1, which makes it fail to resist the misfolding of the protein and significantly disturbs its native structure. Protein misfolding caused by metal dissociation of SOD1 is closely related to the progression of ALS [79]. Copper ions were abnormally accumulated in the spinal cords of patients (SALS: 89.0 ± 57.6 μg/g, n = 7; control: 46.3 ± 28.8 μg/g, n = 12) with sporadic ALS (SALS) and FALS model mice induced by overexpression of the mutant SOD1 gene [80-83]. Interestingly, there were no significant changes in SOD1 levels in the CSF of ALS patients (including SALS and those carrying SOD1 mutations) [84]. Transgenic mice overexpressing human mutant SOD1 have been shown to develop ALS-like symptoms, but not in mice knockout or overexpressing wild-type SOD1 genes, indicating that mutant SOD1 causes motor neuron disease by gaining toxic function rather than by losing normal physiological function [85]. Overall, mutant SOD1 may contribute to the development and progression of ALS by disrupting intracellular copper homeostasis and causing toxicity.

### 4.5 Wilson’s Disease

WD is a rare autosomal recessive disorder of copper metabolism caused by mutations in the ATP7B gene. In addition to clinical symptoms such as chronic hepatitis, cirrhosis, and liver failure, patients may experience neurological or psychiatric symptoms such as tremors, dysarthria, ataxia, Parkinson's syndrome, dystonia, anxiety, and depression. Diagnostic features include corneal pigmentation rings (Kayser-Fleischer rings) due to copper deposition in the cornea, decreased serum ceruloplasmin levels (< 200 mg/L), and elevated urinary copper levels (urinary copper excretion > 100 μg/24 h) [86-89]. In addition, genetic testing and liver biopsy (liver copper content > 250 μg/g dry weight) are also of great diagnostic significance [90]. The liver is the primary organ responsible for maintaining copper homeostasis. In the liver, ATP7B transports copper to the copper-dependent ferroxidase ceruloplasmin and regulates the export of excess copper to bile, which is eventually excreted in the feces [91]. Although ATP7B knockout mice exhibited hepatic copper overload at 6 weeks of age, they did not develop symptoms of WD at this time [92]. In WD, a defect in ATP7B function results in the inability of copper to be incorporated into ceruloplasmin and the dysfunctional excretion of copper into the bile, which ultimately leads to copper accumulation in various organs such as the liver, brain, and cornea, as well as reduced serum levels of ceruloplasmin [9]. Autopsy studies have shown that WD patients (n = 12) have significantly increased copper levels in the cortex (WD: 34.3 ± 17.5 μg/g; control: 3.6 ± 0.9 μg/g) and basal ganglia(WD: 36.6 ± 8.2 μg/g; control: 5.7 ± 0.7 μg/g), an eight-fold increase in brain copper levels compared to controls (WD: 41.0 ± 18.6 μg/g; control: 5.4 ± 1.8 μg/g) (n = 5) [93-95], and liver copper levels that are approximately 25 times higher than normal (WD: 417 ± 83 μg/g, n = 3; normal: 17 ± 9 μg/g, n = 8) [96, 97]. Urinary copper is derived from free copper circulating in the blood. In untreated WD, free copper pools can often be as high as 50 mg/dL, and it is this greatly elevated free copper that contributes to copper toxicity [98]. Furthermore, an excessive copper load leads to tissue failure and death by damaging mitochondria. The mitochondria were found to contain significantly higher copper loads than the nucleus and endoplasmic reticulum (ER) in the liver of WD patients, as well as changes in mitochondrial structure, including increased electron density, separated inner and outer membranes, and giant mitochondria, which were abolished with copper chelators [94].

### 4.6 Menkes Disease

MD is an X-linked recessive, lethal multisystem copper metabolic disease caused by mutations in the ATP7A gene. Most patients are males, and the main clinical features include progressive neurodegeneration, connective tissue abnormalities, and hair abnormalities [99]. As well as regulating neuronal activation and axonal development, ATP7A is essential for copper uptake across the intestinal mucosa and copper transport across the BBB and BCB. ATP7A transports copper to the TGN and delivers it to a series of copper-dependent enzymes [100] while also removing excess copper from the cytosol to maintain intracellular copper levels [101]. In MD, ATP7A dysfunction affects copper transport across the intestinal mucosa and the BBB, resulting in severe systemic copper deficiency [102]. Many symptoms can be attributed to the reduced activity of a range of copper-dependent enzymes, including CCO, tyrosinase, and lysyl oxidase, as demonstrated in animal models and MD patients [99]. However, copper is unevenly distributed in MD patients, with copper accumulating in their intestines and kidneys while significantly lower levels are found in the serum, liver, and brain [103]. In addition, plasma ceruloplasmin levels are lower in MD patients (0.04 g/L; normal range, 0.2-0.6 g/L) [104, 105].

### 4.7 Prion Diseases

Prion diseases (transmissible spongiform encephalopathies, TSEs) are a group of fatal neurodegenerative diseases affecting humans and animals caused by the conformational transition of normal cellular prion protein (PrP^C^) to the pathogenic associated isoform PrP^Sc^ [106]. Prion diseases are characterized by neurological symptoms such as extrapyramidal motor signs, cerebellar ataxia, and myoclonus [107]. Creutzfeldt-Jakob disease (CJD) is the most prevalent human prion disease, accounting for more than 85% of all cases [108, 109]. PrP^C^ is a glycosylphosphatidylinositol (GPI)-anchored plasma membrane protein widely distributed throughout the body and predominantly expressed in the brain (CNS) and exhibits SOD-like activity when bound to copper [110]. PrP^C^ also exhibits copper transporter properties, and its N-terminus contains a highly conserved octapeptide repeat sequence (PHGGGWGQ) with 5-6 Cu^2+^ binding sites [111]. PrP^C^ has a series of physiological functions, such as immunoregulation [112], neuroprotection, antioxidant [113], signal transduction, and synaptic transmission [114]. PrP^Sc^ is a misfolded conformer of PrP^C^ that differs in its structure. PrP^C^ is mainly α-helix in structure, while PrP^Sc^ is rich in β-sheet. PrP^Sc^ aggregates can recruit PrP^C^ protein through a self-perpetuating reaction and convert it to its own conformation [112], and the aggregation and precipitation of protease-resistant PrP^Sc^ is neurotoxic [68]. It has been reported that the copper content in the brain tissue of patients (4.23 ± 0.33 μg/g wet weight, n = 9) with sporadic Creutzfeldt-Jakob disease (sCJD) was reduced by 50% compared with the controls (6.44 ± 0.18 μg/g, n = 3) [115], and there is also a 60% reduction in brain copper levels in scrapie-infected mice, indicating a severe copper deficiency in prion diseases [116]. Furthermore, prion protein (PrP) knockout mice and PrP^Sc^-infected mice had significantly lower copper levels in the brain than wild-type mice [107]. The potential mechanisms for prion neurotoxicity mainly include the loss of PrP^C^ function and the gain of toxic function for PrP^Sc^ [117].

It is interesting to note that there is conflicting evidence regarding the role of copper in prion diseases. On the one hand, PrP requires copper to remain “normal” and non-infectious, and copper deficiency can lead to prion diseases. Copper acts as an antioxidant in copper-containing PrP, thereby enhancing neuronal survival [118]. Copper also inhibits its interaction with PrP^Sc^ by inducing endocytosis of PrP^C^ from the cell surface, thereby reducing the spread of prion diseases [119]. Furthermore, PrP^C^ and copper jointly inhibit N-methyl-D-aspartate receptors (NMDARs), which protect neurons from excitotoxicity [117]. In earlier studies, the copper chelator cuprizone was found to induce clinical symptoms similar to scrapie in mice [120]. According to another study, copper ions delayed the onset of prion diseases in scrapie-infected mice and significantly reduced the accumulation of PrP^Sc^ in scrapie-infected neuroblastoma cells [121]. All of this evidence suggests that copper plays a protective role in prion diseases. On the other hand, the binding of copper to PrP^C^ promotes its conformational transition to PrP^Sc^, increasing protease resistance and protein infectivity [82]. Treatment with the copper chelator D-penicillamine (DPA) delayed disease onset in scrapie-infected mice while reducing copper levels in the blood and brain [119]. In addition, Cu^2+^ is involved in the aggregation of human PrP, mainly through binding His-111, Met-109, and Met-112 to produce neurotoxicity [122]. These pieces of evidence support the role of copper in promoting prion diseases. As a result, copper plays a seemingly paradoxical role in the development of prion diseases, and its mechanism of action is still unclear. Possible reasons are that copper may act differently due to various factors, such as the copper-to-prion ratio, pH, and oxidation state [118].

### 4.8 Multiple Sclerosis

MS is an autoimmune inflammatory disease of the CNS and is the leading cause of neurological disability in young adults [123]. Clinical manifestations include gait disturbance, sensory disturbances, visual impairment, and cognitive deficits [124]. Copper concentrations in serum (MS: 16.44 ± 0.71 μmol/L, n = 29; control: 12.90 ± 1.09 μmol/L, n = 29) and CSF (MS: 0.171 ± 0.02 μmol/L, n = 28; control: 0.088 ± 0.01 μmol/L, n = 28) are significantly elevated in MS patients compared to healthy controls. The possible reason is that the decrease in serum ceruloplasmin activity affects the absorption of copper, leading to increased free copper [24]. Upregulated copper transporters have been found in the CNS of MS patients, and dysregulated copper transport may cause demyelination through astrocytes. In active MS lesions, structural and functional impairment of the BBB can lead to serum copper entry into the CNS, subsequent uptake and release of copper by astrocytes, and induction of demyelination. In contrast, in inactive MS lesions, copper uptake and distribution are also regulated by astrocytes. Notably, restoring copper homeostasis in the white matter may be a potential therapeutic target [125].

## 5 TARGETED DRUGS AND THERAPIES TO REGULATE COPPER HOMEOSTASIS

### 5.1 Metal-protein Attenuating Compounds (MPACs)

MPACs are a class of multifunctional compounds that, unlike traditional chelators, have a moderate affinity for bound metal ions and exhibit mild chelation [128]. They compete mildly with metal ions for target proteins and restore metal homeostasis by regulating metal ion redistribution and disrupting aberrant metal-protein interactions. Therefore, the mechanism of action of MPACs is more complex than chelators and more similar to metal chaperones [129].

Clioquinol (CQ, 5-chloro-7-iodo-8-hydroxyquinoline) is a small hydrophobic molecule that can cross the BBB and has moderate affinity for copper and zinc while simultaneously acting as their bidentate ligand [45]. Oral administration of CQ reduced the Aβ burden in the brains of Tg2576 transgenic mice by 49% and improved cognitive performance [129]. CQ also significantly decreased plasma Aβ_1-42_ concentrations and decreased cognitive deterioration in patients with moderate AD [130]. Furthermore, CQ significantly reduced the loss of SN neurons in mouse models of PD induced by 6-hydroxydopamine (6-OHDA) or MPTP (1-methyl-4-phenyl-1,2,3,6-tetrahydropyridine) [131]. CQ also attenuated neuropathological symptoms in the R6/2 mouse model, an animal model of HD, including HTT accumulation and brain atrophy, and improved motor function [132]. CQ attenuated demyelination, microglial activation, and enhanced autophagy in myelin oligodendrocyte glycoprotein (MOG)-induced experimental autoimmune encephalomyelitis (EAE), one of the most commonly used animal models of MS [133]. As a second-generation MPAC, PBT2 (5,7-dichloro-2-[(dimethylamino)methyl]-8-hydroxyquinoline) is a more effective copper/zinc ionophore than CQ and has higher solubility and BBB permeability [43]. PBT2 significantly reduced the phosphorylated tau and Aβ burden levels in the brain of AD model mice, which effectively improved their synaptic function and learning and memory ability [134]. A clinical trial also indicated that PBT2 markedly reduced CSF Aβ_1-42_ levels and improved cognitive performance in AD patients [129]. PBT2 has a high affinity for Aβ protein. It exerts anti-AD effects by regulating the concentration of metal ions in the brain, especially by effectively removing copper and zinc ions from Aβ [128]. Furthermore, PBT2 significantly improved motor function and prolonged lifespan in R6/2 mice and also decreased paralysis in C. elegans overexpressing an extended polyQ tract [63]. Another clinical trial demonstrated that PBT2 reduced the copper-dependent conversion of mHTT monomers to toxic oligomers [132]. In addition, PBT434 is a novel MPAC with a high affinity for copper, which prevented α-syn accumulation, and protected nigrostriatal dopaminergic circuitry and motor function in animal models of PD [131].

### 5.2 Copper Chelators

Copper chelators are often used to facilitate the excretion of excess copper from the body. DPA, a copper chelator that cannot cross the BBB and has a high affinity for copper ions, ameliorated oxidative stress in AD patients but did not affect cognitive decline [135]. DPA mobilizes copper from the liver and other sites and promotes its excretion into the urine [136], thereby reversing hepatic, neurologic, and psychiatric manifestations in most WD patients [9]. Compared to DPA, the combination of dimercaptosuccinic acid (DMSA) and zinc significantly improved neurologic symptoms in patients with WD [93]. Trientine has a polyamine structure that chelates copper by forming stable complexes with the four constituent nitrogens in a planar ring. It has similar effects to DPA but has fewer side effects and lower risks of neurological deterioration, making it an alternative to DPA for patients who are intolerant to it [90]. Tetrathiomolybdate (TTM) has a lower rate of neurological deterioration (<5%) compared to DPA (50%) and trientine (<20%) [136]. It works through a unique mechanism by forming a stable tripartite complex with copper and protein [98]. In addition, TTM has the advantage of rapid onset, requiring only a few weeks of treatment to restore normal copper balance, and does not increase serum-free copper. In contrast, other copper chelators or zinc take several months [43]. TTM also significantly reduced spinal cord copper ion levels, slowed disease progression, and prolonged lifespan in SOD1^G93A^ mice, exhibiting approximately twice the efficacy of riluzole [85]. Furthermore, combined treatment with CuCl_2_ and the lipophilic copper chelator sodium dimethyldithiocarbamate (DMDTC) significantly increased brain copper content and extended lifespan in MD model mice [102]. PET studies with ^64^Cu indicated that pretreatment of MD model mice with the lipophilic chelator disulfiram increased copper transport to the brain and decreased copper uptake by the kidneys. Combined treatment of copper and disulfiram also ameliorated brain copper deficiency [137]. Zhao *et al.* designed a novel Cu^2+^-specific chelator named TDMQ20, which completely reversed cognitive and behavioral impairments in three different mouse models simulating the early and late stages of AD and inhibited oxidative stress catalyzed by copper-amyloid complexes in the mouse cortex [138].

### 5.3 Copper Supplements, Zinc Salts, and Other Medications

Early parenteral administration of copper-histidine significantly alters the progression of MD, although this approach does not ultimately cure the disease [139]. Zinc preparations are the treatment of choice for WD patients with neurological symptoms and are also effective as maintenance therapies. Unlike chelators, zinc inhibits intestinal copper absorption by inducing MTs in enterocytes and the liver. Zinc intake can increase by 25-fold the expression of MT, which is tightly bound to copper and subsequently shed through the feces, thus preventing copper from entering the bloodstream [140, 141]. Zinc preparations mainly include zinc acetate, zinc sulfate, and zinc gluconate [136]. CuII(gtsm) reduced the abundance of Aβ trimer and phosphorylated tau in APP/PS1 mice, decreased the activity of glycogen synthase kinase-3β (GSK-3β), which mediates neurotoxicity, and ultimately reversed cognitive deficits. In addition, CuII(gtsm) also reduced Aβ levels in APP-CHO cells [142]. The positron emission tomography agent CuII(atsm) selectively localizes to the striatum of PD patients, preventing dopaminergic neuron loss and improving motor impairment. Furthermore, it may also exert a protective effect by modulating copper transmission, copper protein activity, and iron metabolism protein expression. CuII(atsm) restored motor and intestinal dysfunction induced by MPTP and neuronal subpopulations in the myenteric plexus [143]. Furthermore, CuII(atsm) also exerted neuroprotective effects by rescuing nigral cell loss [144], inhibiting α-syn nitration and fibrillation [59]. Interestingly, the copper in CuII(atsm) increased the copper content of mutant SOD1 by transferring into it, resulting in an increase in the pool of fully metallated (holo) SOD1, thereby improving motor function and survival in SOD1^G37R^ mice [145]. Oral administration of CuII(atsm) also significantly increased SOD1 activity in SOD1^G93A^ mice, delayed the onset of paralysis and prolonged lifespan, possibly due to its ability to readily cross the BBB and promote CCS-dependent activation of mutant SOD1 [146]. In addition, CuII(atsm) is an anti-nitrosative agent that exerts a protective effect against ALS neurodegeneration by counteracting protein nitration [29]. The copper ionophore elesclomol has recently been reported to deliver copper to mitochondria and elevate CCO levels in the brain to alleviate detrimental neurodegenerative changes and improve survival in the mottled-brindled mouse, a murine model of severe MD [147].

## 6 POTENTIAL NATURAL COMPOUNDS FOR REGULATING COPPER HOMEOSTASIS

### 6.1 Luteolin

Luteolin is a plant flavonoid extracted from *Elsholtzia rugulosa* (Labiatae) that exhibits several biological effects, including antioxidant, anti-inflammatory, and anti-amnesia. Luteolin plays a neuroprotective role in neurodegenerative diseases and traumatic brain injury (TBI) by inhibiting the activation of immune cells, the release of inflammatory mediators, and neuroinflammatory responses [148]. Researchers have established an *in vitro* model of AD using human neuroblastoma SH-SY5Y cells that overexpress the Swedish mutant form of human APP (APPsw cells), which are induced to become toxic only when copper is added to the culture medium [149]. Luteolin counteracted the toxicity mediated by copper in APPsw cells by down-regulating the expression of amyloid-β protein precursor (AβPP), inhibiting the secretion of Aβ_1-42_, inhibiting apoptosis, and regulating redox imbalance [150]. Another study indicated that the coordination and transfer of Cu^2+^ significantly increased the free radical scavenging efficiency and antioxidant activity of luteolin [151].

### 6.2 Apigenin

Apigenin is a less toxic flavonoid derived from herbs such as *Carduus crispus* and *Elsholtzia rugulosa* [152]. It has biological effects such as antioxidant, anticancer, neuroprotective, and free radical scavenging. Apigenin antagonized copper-mediated Aβ neurotoxicity in APPsw cells and exerted neuroprotective effects mainly by alleviating oxidative stress, inhibiting ROS-induced p38 mitogen-activated protein kinases (p38 MAPK) and stress-activated protein kinase (SAPK)/c-Jun N-terminal kinase (JNK) signaling pathways, inhibiting apoptosis and protecting mitochondrial function [153]. Interestingly, luteolin, apigenin, and rosmarinic acid (RA) can be simultaneously isolated from the copper-tolerant plant *Elsholtzia splendens*, a native Chinese herb of the Labiatae family grown in copper deposits in China [154].

### 6.3 Vitegnoside

Vitegnoside is a flavonoid derived from *Vitex negundo*, which is used as a folk medicinal plant in Asian countries such as China and Japan and has biological activities such as antioxidant, anti-inflammatory, and anti-osteoporosis. By inhibiting the p38 MAPK/JNK signaling pathway, vitegnoside attenuated neuronal injury, inflammation, and mitochondria-mediated apoptosis in copper-induced APPsw cells [155].

### 6.4 Quercetin

Quercetin is a natural plant flavonol with a polyphenol structure, mainly derived from red onion, cranberry, and blueberry. It also exists in herbal plants such as *Hypericum perforatum*, *Ginkgo biloba*, and elderberry. Quercetin has biological activities such as antioxidant, anti-ischemic, anti-inflammatory, and anti-cancer, and is widely used in the treatment of neurological disorders, tumors, and cardiovascular diseases [156-158]. For example, studies have shown that it exerts significant neuroprotective effects in TBI [158]. Quercetin significantly inhibited the formation of OH^-^ in the Fenton reaction by chelating with copper through Electron Paramagnetic Resonance (EPR) spin-trapping experiments. In addition, the Cu^2+^-quercetin complex exhibited stronger free radical scavenging activity [159]. Quercetin inhibited copper-induced oxidative stress in SH-SY5Y cells and attenuated copper-induced apoptosis and ER stress by upregulating autophagosome-bound microtubule-associated protein light chain-3 II (LC3II) to regulate autophagy [160]. It was discovered in another study that quercetin also exerted neuroprotective effects on copper-induced P19 neurons by regulating the phosphatidylinositol-3-kinase (PI3K)/protein kinase B (Akt) and ERK1/2 signaling pathways [161]. In addition, quercetin enhanced the induction of MT by copper in a dose-dependent manner, consistent with the mechanism of action of zinc preparations for treating WD, revealing the remarkable potential of quercetin in controlling copper toxicity in WD [162].

### 6.5 Epigallocatechin Gallate (EGCG)

The main bioactive components of green tea (*Camellia sinensis*) are catechins, of which EGCG is the most abundant. EGCG is a flavone-3-ol polyphenol with a remarkable ability to scavenge free radicals and particularly prominent anticancer effects [163, 164]. EGCG exerts neuroprotective effects in models of AD, PD, ALS, and ischemic stroke [165]. EGCG reduced Cu^2+^-induced ROS production in α-syn-transduced PC12 cells, inhibited the overexpression and fibrillation of α-syn, and protected cells from Cu^2+^-mediated toxicity. In addition, EGCG also inhibited the Cu^2+^-induced conformational transition of α-syn to β-sheet by coordinating with Cu^2+^ to form a Cu^2+^-EGCG complex, and this complex also inhibited Cu^2+^-induced α-syn fibrillation [166].

### 6.6 Myricetin

Myricetin is a natural flavonoid extracted from several herbs, such as *Ginkgo biloba* and *St. John’s Wort*, which has therapeutic effects on CNS diseases such as AD, PD, and depression [167-169]. Myricetin inhibited the formation of Aβ aggregates by competing with Aβ for binding metal ions and reduced the neurotoxicity of Aβ in Cu^2+^ and Zn^2+^-treated human neuroblastoma SK-N-BE(2)-M17 cells (M17) [170, 171].

### 6.7 Curcumin

Curcumin is a hydrophobic polyphenol in the herbal plant *Curcuma longa* (Zingiberaceae) with anti-inflammatory, antioxidant, and antidepressant bioactivities [172], and is widely used in the treatment of AD, depression, cancer, and various inflammatory diseases [173]. Curcumin reduced anxiety caused by subchronic copper toxicity by modulating serotonin and improved spatial learning and memory abilities [174]. Furthermore, liposome-encapsulated curcumin (LEC) significantly attenuated copper-induced liver injury and liver fibrosis in an ATP7B-knockout WD mouse model [175]. Dendrosomal nano-curcumin (DNC) exerted antioxidant and anti-inflammatory effects in the EAE model of MS, also resisted cuprizone-induced toxic demyelination, and effectively protected myelinating cells [176]. Low doses of curcumin inhibited Cu^2+^-induced oxidative stress and reversed neuronal damage in rat cortical neurons [177]. Furthermore, nano-curcumin has similar activity to curcumin but has higher solubility and stability. They attenuated CuSO_4_-induced neurotoxicity by alleviating oxidative damage, apoptosis, and inflammatory responses and modulating the Akt/GSK-3β pathway [178]. It has been demonstrated that curcumin can protect the dopaminergic system and motor performance against subchronic and acute copper toxicity [144, 179], which is consistent with the neuroprotective effects exerted by AEAAG against copper toxicity. *In vitro* studies showed that curcumin attenuated copper-induced neurotoxicity in SH-SY5Y cells by inhibiting mitochondrial apoptosis and oxidative stress [171]. It also significantly inhibited Cu^2+^-induced upregulation of APP and BACE1 transcript levels in PC12 cells [180].

### 6.8 Rutin

Rutin is a glycoside composed of the flavonol aglycone quercetin and disaccharide rutinose derived from the herbal plant *Ruta graveolens* and fruits such as oranges, grapes, and berries [181]. Like most polyphenols, rutin crosses the BBB and exhibits antioxidant properties. Rutin ameliorated copper-induced brain damage, including perforated laminae of the cerebral cortex and neuronal degeneration, by alleviating oxidative stress and neuroinflammation [182].

### 6.9 Resveratrol

Resveratrol is a natural polyphenol compound present in various plants, including *rhizoma polygonum cuspidatum*, grapes, pine nuts, and peanuts, and also rich in red wine. In addition to its antioxidant and anti-inflammatory properties, it has been shown to improve cerebral ischemic injury and cardiovascular disease [183, 184]. Resveratrol modulated plasma copper and zinc levels and affected oxidative stress and antioxidant status in copper-deficient rats [185]. In addition, it attenuated CuSO_4_-induced senescence by up-regulating autophagy to regulate cellular proteostasis [186]. Resveratrol also modulated the homeostasis of copper and zinc levels in the blood and improved oxidative stress in a rat model of Type 2 diabetes [187] and aluminum exposure [188]. Dietary supplementation with resveratrol improved plasma copper and zinc levels, antioxidant status, lipid metabolism, and vasodilation in copper-induced (CuNPs/CuCO_3_) mice [189]. Another study found that resveratrol effectively alleviated oxidative stress induced by copper oxide nanoparticles (CuONPs) and liver and kidney damage [190].

## CONCLUSION

A growing body of evidence, including preclinical studies *in vivo* and *in vitro*, meta-analyses, and large epidemiological studies, suggests that one of the potential risk factors for neurodegenerative diseases is an age-related imbalance in metal homeostasis. Copper is an important biologic metal, present in high micromolar concentrations in cortical tissue and released as a free ion during neural activity. The trace element copper plays a vital role in maintaining the body's growth, development, and metabolic homeostasis. Excessive copper loading leads to oxidative damage through the Fenton reaction, while copper deficiency disrupts its normal physiological function by affecting copper enzyme activity. Abnormal copper metabolism or distribution may lead to a variety of diseases, with a particular link to neurodegenerative diseases. In the past few decades, people's average life expectancy has gradually increased, and the incidence of age-related neurodegenerative diseases has been rising globally, imposing a heavy burden on society and patients. Currently, drugs that aim to regulate copper balance are mainly a series of MPACs, copper chelators, copper supplements, and zinc salts. However, these treatments can only temporarily alleviate the disease's symptoms to a certain extent but cannot prevent or reverse its progression. In addition, many drugs have limitations, including limited therapeutic efficacy, severe side effects, and poor compliance. For example, chronic or excessive intake of CQ resulted in severe deficiencies of copper, zinc, and iron and subacute myelo-optic neuropathy (SMON), which was previously prevalent in the Japanese population [191]. The early adverse effects of DPA include fever, rash, proteinuria, lymphadenopathy, and thrombocytopenia, while the late side effects include nephrotoxicity and bone marrow suppression [90]. TTM also has side effects such as anemia, leukopenia, and elevation of transaminase enzymes [136]. Even though zinc salts have been reported to cause fewer side effects, mainly gastric problems [192], they are very slow-acting, and free copper is not expected to be controlled within 6-12 months, during which time the disease may continue to progress [98]. Furthermore, finding the right timing for treatment is also challenging. Copper-histidine subcutaneous injections are effective depending on the maturity of the BBB and residual copper transport activity, with the maturity of the BBB determining whether the copper is captured or delivered to the neurons. Therefore, copper injection has shown promising clinical efficacy in neonates with immature BBB but limited therapeutic effect in patients older than two months old [137]. Furthermore, the absence of overt clinical signs and reliable biochemical markers makes it difficult to distinguish neonates with MDs from healthy neonates [193]. Therefore, there are significant difficulties with newborn screening and early treatment of MD. Notably, both CQ and PBT2 failed in phase II trials, possibly due to their inability to distinguish copper bound to essential metalloproteins from toxic copper bound to Aβ [138]. DPA cannot directly alter intracellular copper homeostasis since it is difficult to cross the BBB and has limited therapeutic effect [194].

In addition to drugs to treat neurodegenerative diseases, gene therapy is also promising. For example, silencing CTR1 inhibited α-syn aggregation caused by intracellular copper accumulation [195], overexpression of MT-3 significantly reduced polyQ aggregation and toxicity in an HD cell model [64], and the addition of ATP7A gene targeting the brain *via* an AAV-5 vector increased survival in mouse models of MD [15]. It is also worth noting that copper overload has been found in many neurodegenerative diseases. In contrast, the copper chelator TTM effectively inhibits cuproptosis, and the metabolite α-lipoic acid in the cuproptosis pathway also exhibited substantial potential in both *in vitro* and *in vivo* models of WD [196]. Therefore, it is reasonable to speculate that cuproptosis may play an important role in the progression of neurodegenerative diseases. Cuproptosis is likely to become another potential critical therapeutic target for neurodegenerative diseases. In addition, the risk of excessive copper exposure can also be reduced by installing reverse osmosis devices on faucets, avoiding copper-containing supplements, reducing meat intake appropriately, and avoiding occupational activities with long-term exposure to metals [13, 197]. Although some existing drugs can reduce pathological copper deposition to a certain extent, they cannot restore normal copper metabolism. To date, liver transplantation remains the only option for permanently restoring normal copper metabolism [198]. However, the shortage of donors and the need for lifelong immunosuppressive medication significantly limit its application [199]. Therefore, developing novel, safe, effective drugs remains an urgent problem, and modulation targeting copper levels remains a promising option. The drug should at least meet some basic requirements, including the ability to cross the BBB, sound therapeutic effects, no or only minimal toxic side effects, and not chelating other essential trace metals.

Natural compounds represent a valuable cultural legacy of humanity. Numerous studies have pointed out the neuroprotective effects of many natural compounds. For example, icariin is a flavonoid compound derived from the genus *Epimedium*, which effectively improved the neuroethology function of TBI rats and reduced neuroinflammation and pathological injury [200]. Many medicinal plants and their natural components have antioxidant, free radical scavenging, and neuroprotective pharmacological properties and have a remarkable effect in preventing copper-induced neurotoxicity. *Dracocephalum moldavica* L. (Labiatae) is a traditional herbal medicine mainly produced in northern China and has substantial medicinal value for diseases such as hypertension, coronary heart disease, and hepatitis. In copper-injured APPsw cells, total flavonoid extracts of *Dracocephalum moldavica L.* reduced copper-induced toxicity by modulating redox imbalance, APP expression, Aβ_1–42_ content, and the extracellular signal-regulated kinase (ERK)/ cAMP response element-binding protein (CREB)/brain-derived neurotrophic factor (BDNF) pathway [149]. *Aloe arborescens Miller* (Liliaceae) has various pharmacological effects, including antioxidant, anti-inflammatory, and anti-cancer properties. It was found that its extract, Aloe arborescens gel (AEAAG), reversed locomotor deficits in rats with acute copper toxicity. Furthermore, AEAAG reversed the loss of tyrosine hydroxylase (TH) expression within SNpc, ventral tegmental area (VTA), and the subsequent striatal outputs. The neuroprotective effect of AEAAG against copper-induced dopaminergic neurotoxicity reveals its potential in preventing heavy metal neurotoxicity and treating PD [201]. EGb761 is a standardized extract derived from *Ginkgo biloba* leaves, containing mainly ginkgo flavonoid glycosides, terpene trilactones, ginkgolides, and bilobalide, which has neuroprotective effects against AD, PD, and subarachnoid hemorrhage (SAH) [202]. Rojas *et al.* used EGb761 to pretreat MPP^+^ (1-methyl-4-phenylpyridinium, the active metabolite of MPTP)-induced PD mouse model. They found that copper content in the corpus striatum of mice treated with MPP^+^ was significantly decreased, whereas copper content in the midbrain and hippocampus increased significantly. EGb761 pretreatment prevented changes in copper content in these brain regions, suggesting that EGb761 blocked MPP^+^ neurotoxicity by modulating copper homeostasis in the brain [203]. *Kaempferia galanga* L. (Zingiberaceae) has pharmacological activities such as antimicrobial, anti-inflammatory, analgesic, sedative, and antiparasitic [173], and kaempferol is a flavonoid mainly derived from its rhizome [204], which exerted a certain protective effect on DNA damage mediated by singlet oxygen, OH^-^ and superoxide radical anions. In comparison to free kaempferol, Cu-kaempferol complexes exhibited stronger free radical scavenging effects. The antioxidant properties of kaempferol under copper-Fenton conditions suggest that it may exert neuroprotective effects in neurological diseases involving disturbances in copper metabolism [205]. RA is a polyphenolic compound first isolated from rosemary. It exists in many medicinal herbs of the Boraginaceae family and Nepetoideae sub-family of the Lamiaceae family, including *Perilla frutescens* and *Melissa officinalis* [206]. RA exerted a protective effect by interfering with the anomalous and toxic Cu^2+^-Aβ interaction. Furthermore, RA binds Cu^2+^ and mediates the interaction between Aβ and the paramagnetic ion. It also weakly interacts with Aβ and reduces its mediated cytotoxicity [207]. Astragaloside IV (AS-IV) is a triterpenoid saponin present in the root of *Astragalus membranaceus*, which has anti-apoptosis, anti-oxidation, and anti-inflammatory properties, and exhibits neuroprotective effects on AD, PD, and cerebral ischemia [208]. Another study established an *in vivo* specific Cu(I) reporting system, P_MT1F_-EGFP reporter, and found that AS-IV significantly increased intracellular copper ions, even in the low-copper microenvironment [209]. In addition to natural herbs, synthetic herbal derivatives, such as naringin, have shown great potential in the treatment of neurodegenerative diseases. Naringin is a natural flavonoid in citrus fruits such as *Citrus aurantium* L. (*Fructus aurantia*) and *Drynaria fortunei* (Kunze) J. Sm [210-212]. A study has synthesized N,N’-1,10-bis(naringin) triethylenetetraamine bis-Schiff bases as a Cu^2+^ chelator. It effectively inhibited Cu^2+^-induced Aβ_1-42_ aggregations and reduced the toxicity mediated by Aβ_1-42_-Cu^2+^ in PC12 cells by inhibiting ROS production and enhancing superoxide dismutase (SOD) activity [213]. The design and synthesis of natural compounds and metal-chelating agents have also opened up a new field for developing new drugs. A novel multi-target compound has been synthesized by combining resveratrol with the pharmacophore moiety of CQ. This hybrid combines the characteristics of resveratrol and CQ simultaneously. It can cross the BBB, significantly inhibit Cu^2+^-induced Aβ aggregation, and could also control the production of OH^-^ triggered by Cu^2+^, exhibiting excellent multi-target-directed ligand (MTDL) properties [214, 215]. Natural compounds are important sources for drug development and offer promising applications for the prevention and treatment of neurodegenerative diseases. There is great potential for the future development of new drugs for the treatment of neurodegenerative diseases based on the regulation of copper metabolism from natural compounds, and it deserves in-depth study.

## Figures and Tables

**Table 1:** Summary of the copper-related genes, pathogenesis, and changes in copper homeostasis in neurodegenerative diseases.

**Diseases**	**Genes**	**Copper-related Pathogenesis**	**Changes in Copper Homeostasis**		**References**
AD	ApoE4, ATP7B	Copper promotes Aβ and tau aggregation, oxidative damage, and neuroinflammation and inhibits Aβ clearance by affecting the metabolism of BACE1, APP, Aβ, tau, and LRP1.	Serum Cu Brain ceruloplasmin Brain Cu (while in Aβ↑)	↑ ↑ ↓	[13, 26, 40-46, 48-55, 126]
PD	-	Copper promotes α-syn aggregation, affects redox homeostasis, and causes damage to dopaminergic neurons by binding to α-syn and regulating iron metabolism.	Caudate nucleus Cu SN Cu	↓ ↓	[46, 59, 61]
HD	-	Copper promotes HTT aggregation and affects its conformation, increases the aggregation and toxicity of polyQ, and inhibits LDH activity to result in neurodegeneration.	Brain Cu (particularly in the putamen and SN)	↑	[45, 64-69]
ALS	SOD1	Mutations in SOD1 result in defective binding to Cu and protein misfolding, disrupting intracellular copper homeostasis and resulting in toxicity.	Spinal cord Cu Motor cortex Cu Serum Cu and ceruloplasmin	↑ ↑ ↓	[27, 45, 72-75, 78-83, 85, 127]
WD	ATP7B	Defective ATP7B function results in the inability of copper to be incorporated into ceruloplasmin, the dysfunction of copper excretion into the bile, and the disruption of mitochondrial function by the excess copper load.	Liver, brain and cornea Cu Urine Cu Serum ceruloplasmin	↑ ↑ ↓	[9, 86-90, 93-98]
MD	ATP7A	Mutations in the ATP7A gene affect copper transport across the intestinal mucosa and the BBB, resulting in decreased activity of a series of copper-dependent enzymes and systemic copper deficiency.	Intestine, kidney Cu Serum, liver, and brain Cu Plasma ceruloplasmin	↑ ↓ ↓	[99, 102-105]
Prion diseases	-	Copper binding to PrP^C^ promotes the conformational transition of PrP^C^ to PrP^Sc^, increases protease resistance and protein infectivity, and Cu^2+^ is also involved in the aggregation of human PrP.	Brain Cu	↓	[82, 107, 115, 116, 122]
MS	-	Dysregulation of copper homeostasis causes demyelination by astrocytes.	Serum Cu CSF Cu	↑ ↑	[24, 125]

**Table 2:** Summary of *in vitro* studies of natural compounds with the potential to improve neurodegenerative diseases by modulating copper metabolism.

**Compounds**	**Chemical Structure**	**Models**	**Dosage**	**Pharmacological Effects**	**Molecular Mechanisms**	**References**
Luteolin	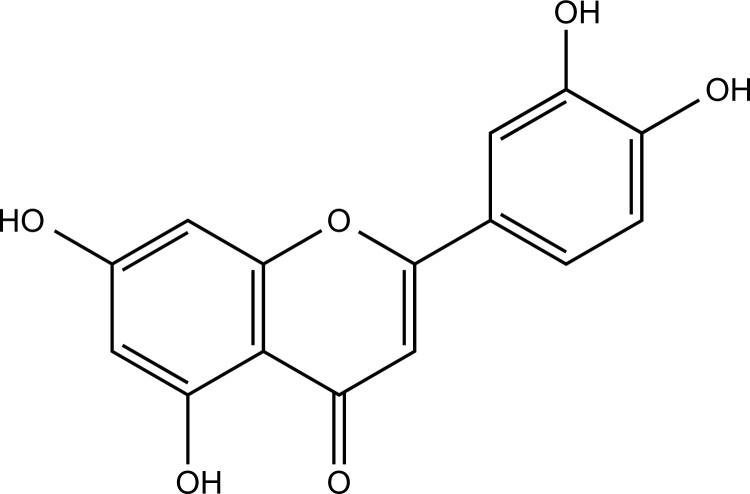	Copper-induced APPsw cells	1, 10 μM for 24 h	Increase cell viability, inhibit Aβ secretion and apoptosis, regulate redox imbalance, and protect mitochondrial function.	Downregulate AβPP expression; reduce intracellular ROS production; enhance SOD activity; reverse mitochondrial membrane potential dissipation; inhibit the activities of caspase-3 and caspase-9.	[150]
Apigenin	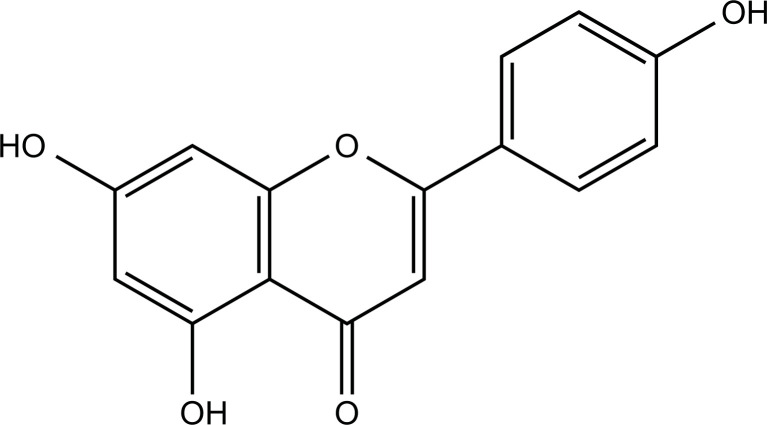	Copper-induced APPsw cells	0.1, 1, 10 μM for 24 h	Alleviate oxidative stress, inhibit neuronal apoptosis, increase neuronal viability, relieve mitochondrial membrane dissipation and neuronal nuclear condensation.	Increase GSH levels; enhance GSH-Px and SOD activities; reduce ROS production; inhibit ROS-induced p38 MAPK-MK2-Hsp27 and SAPK/JNK signaling pathway; inhibit cytochrome c release and caspase-3, caspase-9 activity.	[153]
Vitegnoside	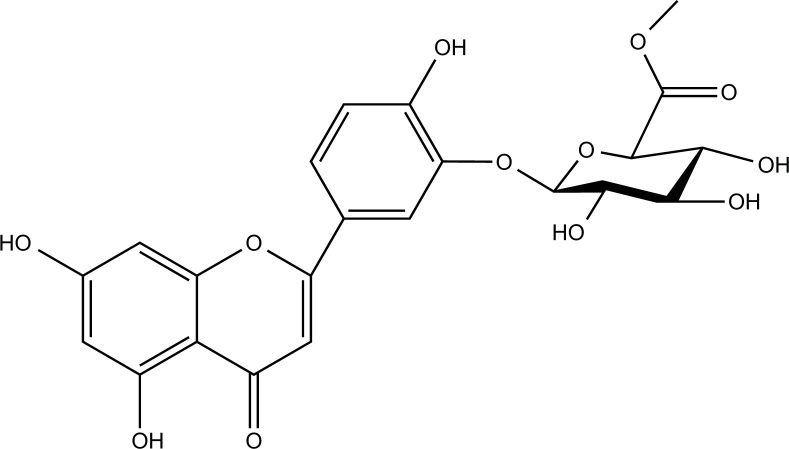	Copper-induced APPsw cells	0.3, 1, 3, 10, 30 μM	Improve cell viability, protect mitochondrial function, inhibit inflammation and mitochondrial-mediated apoptosis.	Inhibit cytochrome c release; reduce caspase-3 and caspase-9 activation; reduce Bax/Bcl_2_ ratio; inhibit p38 MAPK and JNK signaling pathways.	[155]
Quercetin	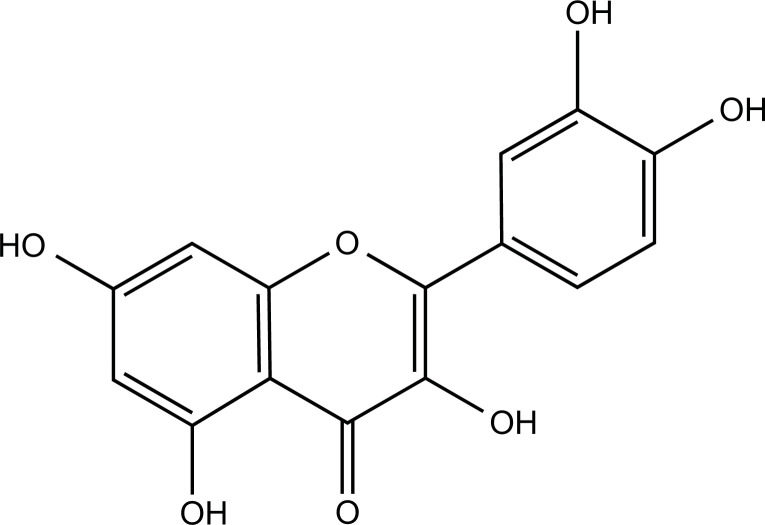	CuSO_4_-induced SH-SY5Y cells	50 nm for 4 h	Improve cell viability, inhibit oxidative stress and ER stress, regulate autophagy to inhibit apoptosis.	Inhibit intracellular ROS levels; restore mitochondrial membrane potential; upregulate Bcl_2_ and LC3II expression; downregulate Bax, cleaved PARP, cleaved caspase 3, cytochrome c, p53, XBP1, PERK, NRF2, CHOP, BiP, Bim, caspase 12 expression and α-syn levels.	[160]
		CuSO_4_-induced P19 neurons	3, 30, 150 μM for 24 h	Improve neuronal survival, inhibit oxidative injury.	Inhibit ROS production, chromatin condensation and decreased caspase-3/7 activity; reduce increased PUMA expression, upregulate NME1; regulate PI3K/Akt and ERK1/2 signaling.	[161]
EGCG	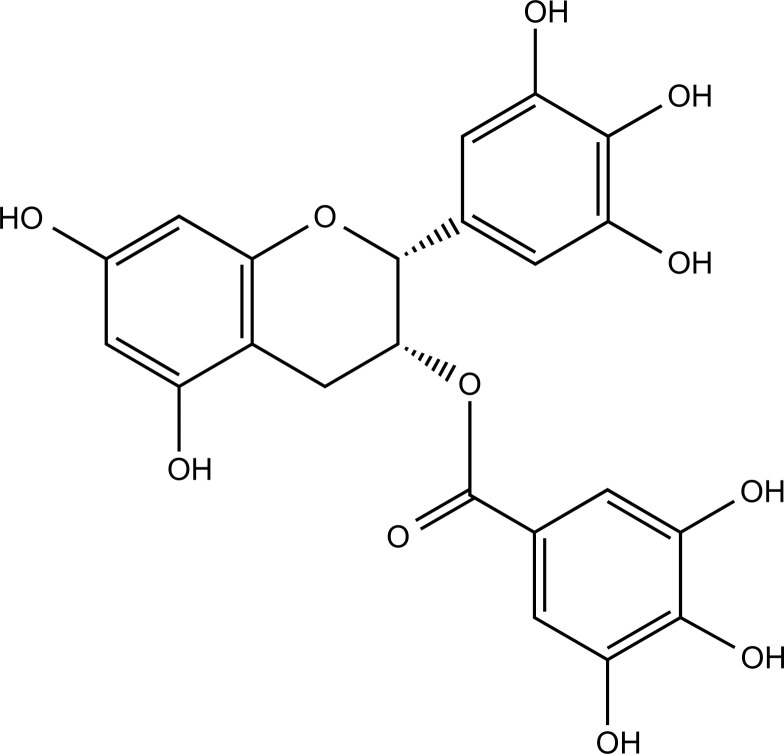	α-syn gene transduced PC12 cells	1, 5, 10, 20, 30 µM for 48 h or 1, 2, 5, 10, 20 µM for 24 h	Inhibit the production of α-syn, reduce cell death and oxidative stress.	Hinder α-syn conformation transition and ROS production; reduce α-syn overexpression and fibrillation.	[166]
Myricetin	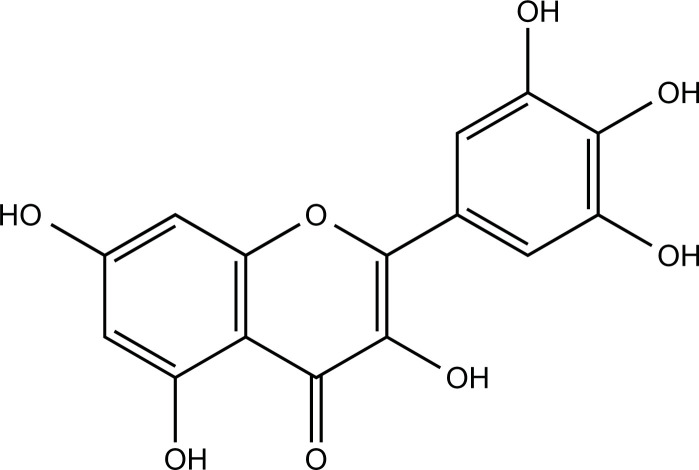	Aβ, CuCl_2_ and ZnCl_2_ induced SK-N-BE(2)-M17 cells	50 μM for 4 or 24 h, 20 μM for 24 h	Inhibit the formation of Aβ aggregates, reduce copper-induced Aβ cytotoxicity, and improve the cell survival rate.	Compete with Aβ to bind copper ions.	[170]
Curcumin	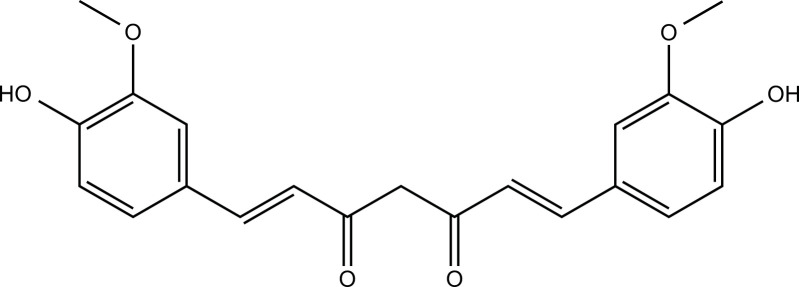	CuSO_4_-induced SH-SY5Y cells	1, 2.5, 5 μM for 3 h	Inhibit oxidative stress and mitochondrial apoptosis.	Downregulate ROS, MDA levels, and Bax/Bcl_2_ ratio; increase SOD and CAT activities; attenuate mitochondrial membrane potential decline and nuclear translocation of cytochrome c; upregulate pro-caspase-3, pro-caspase-9, and PARP1 levels.	[171]

**Table 3:** Summary of *in vivo* studies of natural compounds with the potential to improve neurodegenerative diseases by modulating copper metabolism.

**Compounds**	**Chemical Structure**	**Models**	**Dosage**	**Pharmacological Effects**	**Molecular Mechanisms**	**References**
Curcumin	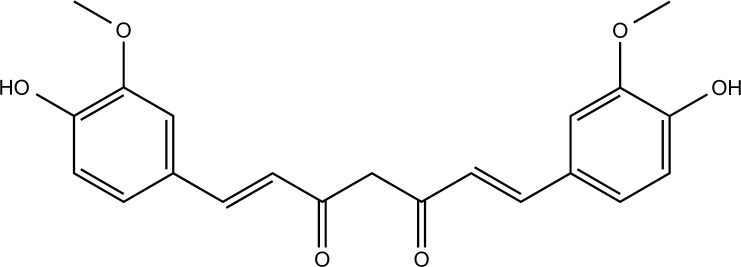	CuSO_4_-induced Wistar rats	80 mg/kg orally for 7 days	Attenuate oxidative injury, apoptosis, and inflammation.	Downregulate brain levels of MDA, NF-κB p65, TNF-α, IL-6, Bax, p53, and caspase-3; increase brain levels of GSH, SOD, CAT, Bcl-2, and BDNF; reduce DNA fragmentation; increase brain AKT and GSK-3β phosphorylation.	[178]
		Copper-induced subchronic copper intoxication in Wistar rats	30 mg/kg orally for 6 weeks	Improve locomotor performance.	Reverse the loss of GFAP and TH expression in SNpc, VTA, and the subsequent striatal outputs.	[179]
		Copper-induced acute copper intoxication in Wistar rats	30 mg/kg orally for 3 days	Enhance locomotor performance.	Reverse the loss of TH expression in SNpc, VTA, and the subsequent striatal outputs.	[144]
Rutin	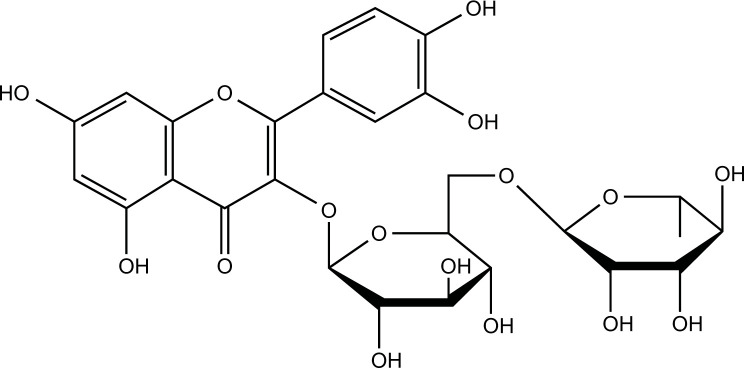	CuSO_4_-induced Wistar rats	100 mg/kg orally for 7 weeks	Antioxidant, inhibit inflammation.	Increase SOD, CAT, GPx, GSH, and AchE activities; decrease MPO activity, NO levels, COX-2, and iNOS expression.	[182]
Resveratrol	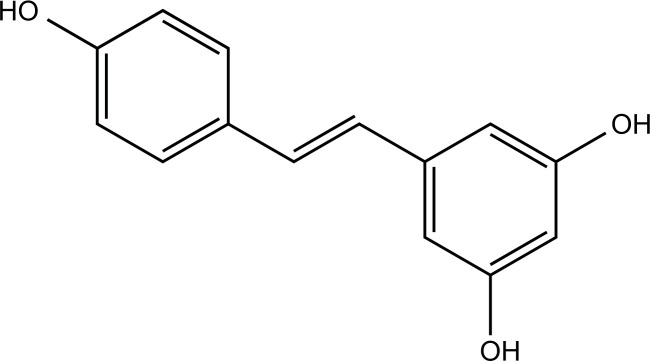	CuCO_3_/CuNPs-induced Wistar rats	500 mg/kg in diet orally for 8 weeks	Improve vascular responses, the lipid profile, and the antioxidant mechanism.	Increase plasma SOD activity and zinc levels; decrease plasma Cu, CAT, GPx, LDL-c, LOOH, and MDA levels; decrease fasting blood glucose; regulate vasodilation.	[189]
		CuONPs-induced Wistar rats	60 mg/kg i.g. for 7 days	Improve liver and kidney pathological changes.	Reduce serum TOS, TAC, creatinine, and urea levels; suppress the elevation of ALT and AST levels.	[190]

## LIST OF ABBREVIATIONS

AD: Alzheimer's Disease
AEAAG: Aloe Arborescens Gel
Akt: Protein Kinase B
ALS: Amyotrophic Lateral Sclerosis
ApoE: Apolipoprotein E
ApoE4: Apolipoprotein E4
APP: Amyloid Precursor Protein
AS-IV: Astragaloside IV
ATOX1: Antioxidant Protein 1
ATP7A: P-type Copper-transporting Atpase α
ATP7B: P-type Copper-transporting Atpase β
Aβ: Amyloid β
AβPP: Amyloid-β Protein Precursor
α-syn: α-synuclein
BACE1: β-site APP Cleaving Enzyme 1
BBB: Blood-brain Barrier
BCB: Blood-cerebrospinal Fluid Barrier
BDNF: Brain-derived Neurotrophic Factor
CAG: Cytosine-Adenine-Guanine
CCO: Cytochrome C Oxidase
CCS: Copper Chaperone for Superoxide Dismutase
CJD: Creutzfeldt-Jakob Disease
CNS: Central Nervous System
COX17: Copper Chaperone for CCO
Cp: Ceruloplasmin
CQ: Clioquinol
CREB: Camp Response Element-binding Protein
CSF: Cerebrospinal Fluid
CTR1: Copper Transporter 1
Cu: Copper
Cu/Zn SOD or SOD1: Copper/Zinc-superoxide Dismutase
Cu^+^: Organic Copper
Cu^2+^: Inorganic Copper
CuONPs: Copper Oxide Nanoparticles
DMDTC: Dimethyldithiocarbamate
DMSA: Dimercaptosuccinic Acid
DMT1: Divalent Metal Transporter 1
DNA: Deoxyribonucleic Acid
DNC: Dendrosomal Nano-curcumin
DPA: D-penicillamine
EAE: Experimental Autoimmune Encephalomyelitis
EGCG: Epigallocatechin Gallate
EPR: Electron Paramagnetic Resonance
ER: Endoplasmic Reticulum
ERK: Extracellular Signal-regulated Kinase
FALS: Familial ALS
Fe-S: Iron-sulfur
GPI: Glycosylphosphatidylinositol
GSH: Glutathione
GSK-3β: Glycogen Synthase Kinase-3β
H_2_O_2_: Hydrogen Peroxide
HD: Huntington's Disease
HTT: Huntingtin
JNK: c-Jun N-terminal Kinase
LC3II: Microtubule-associated Protein Light Chain-3 II
LDH: Lactate Dehydrogenase
LEC: Liposome-encapsulated Curcumin
LRP1: Low-density Lipoprotein Receptor-related Protein-1
MCI: Mild Cognitive Impairment
MD: Menkes Disease
mHTT: Mutant Huntingtin
MOG: Myelin Oligodendrocyte Glycoprotein
MPACs: Metal-Protein Attenuating Compounds
MPP^+^: 1-Methyl-4-phenylpyridinium
MPTP: 1-Methyl-4-phenyl-1,2,3,6-tetrahydropyridine
MS: Multiple Sclerosis
MT: Metallothionein
MTDL: Multi-target-directed Ligand
NMDARs: N-methyl-D-aspartate Receptors
Non-Cp-Cu: Non-ceruloplasmin-bound Copper
6-OHDA: 6-Hydroxydopamine
O2^-^: Superoxide Anions
OH^-^: Hydroxyl Radicals
p38 MAPK: P38 Mitogen-activated Protein Kinases
PBT2: 5,7-dichloro-2-[(dimethylamino)methyl]-8-hydroxyquinoline
PD: Parkinson's Disease
PI3K: Phosphatidylinositol-3-kinase
polyQ: Polyglutamine
PrP: Prion Protein
RA: Rosmarinic Acid
RNS: Reactive Nitrogen Species
ROS: Reactive Oxygen Species
SAH: Subarachnoid Hemorrhage
SALS: Sporadic ALS
SAPK: Stress-activated Protein Kinase
SCJD: Sporadic CJD
SMON: Subacute Myelo-optic Neuropathy
SN: Substantia Nigra
SNpc: Substantia Nigra Pars Compacta
SOD: Superoxide Dismutase
STEAP: Six Transmembrane Epithelial Antigen of The Prostate
Tau: Tubulin-associated Unit
TBI: Traumatic Brain Injury
TCA: Tricarboxylic Acid
TCM: Traditional Chinese Medicine
TGN: Trans-golgi Network
TH: Tyrosine Hydroxylase
TNF-α: Tumor Necrosis Factor-α
TTM: Tetrathiomolybdate
VTA: Ventral Tegmental Area
WD: Wilson's Disease
